# Supplementary figures and images for: Squeezed from the top: “Social Outburst” (2019) and elite overproduction. A study of the dynamics of Chilean political instability from the approach of Structural Demographic Theory
Source: PLoS One. 2024 Jun 13;19(6):e0299063. doi: 10.1371/journal.pone.0299063 (PMC11175532; doi:10.1371/journal.pone.0299063)

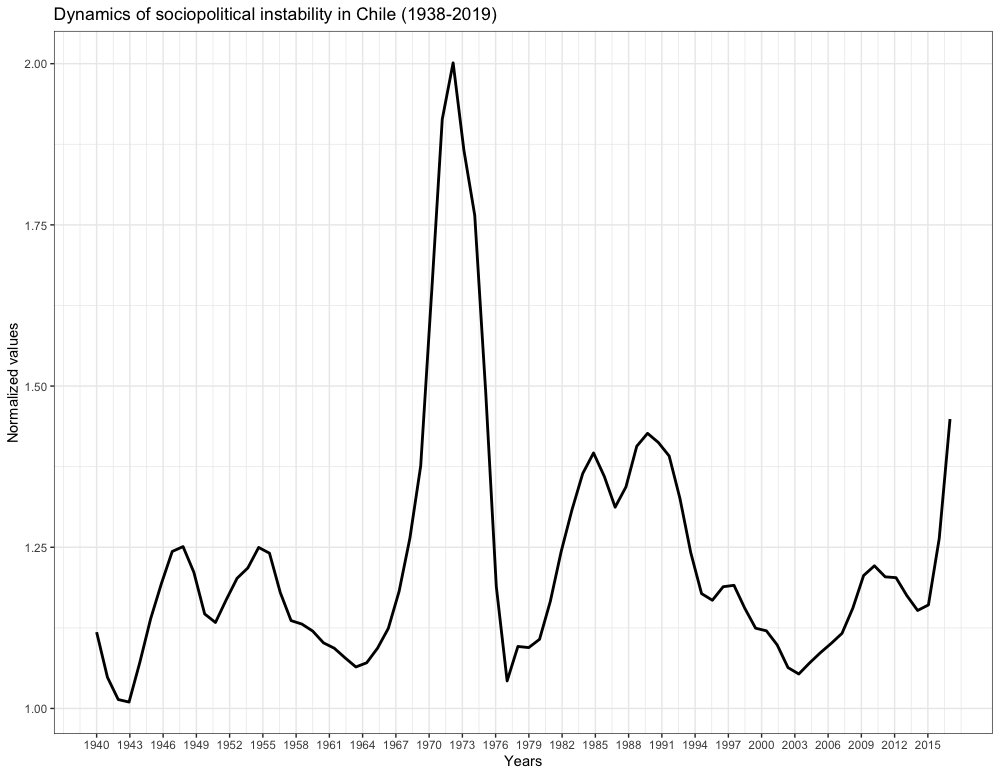

Supplement: S1 Fig — (TIF) [file pone.0299063.s001.tif]

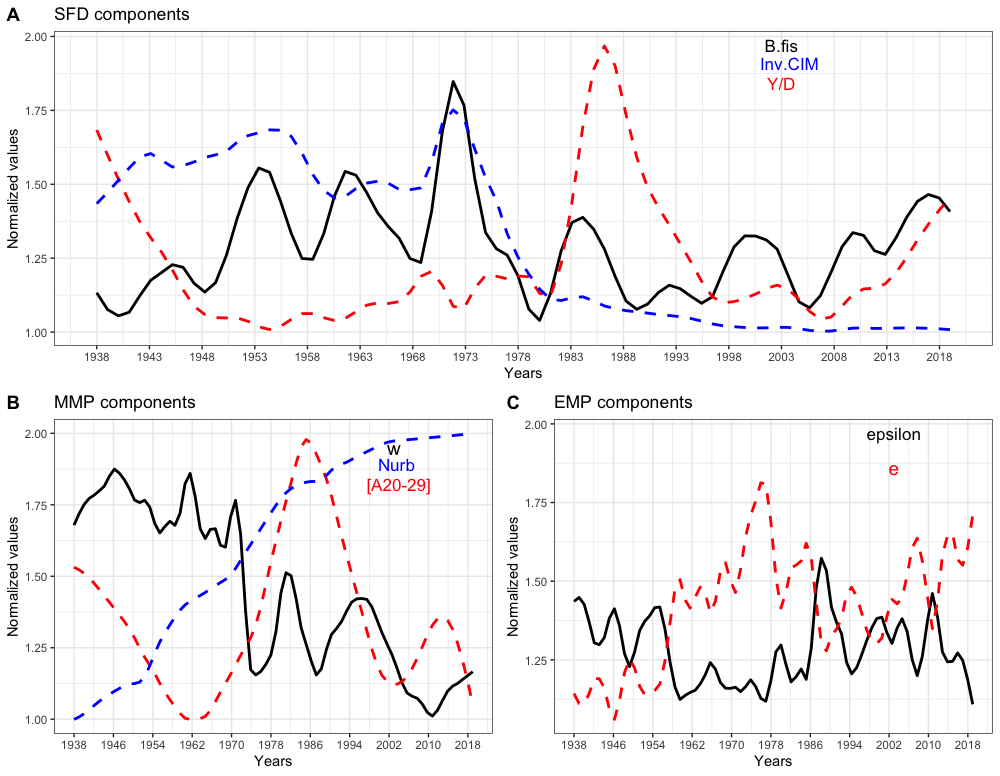

Supplement: S2 Fig — A) SFD components: Budgetary income and expenses (B.fis, black), ratio between public debt and gross domestic product (Y/G,red dotted series) and inverse of intensive contract money index (Inv.CIM, blue dotted series). B) MMP components: Median relative income of the population (w, black series), relative urban population (Nurb, blue series) and proportion of population aged 20–29 ([A20-29], red series). C) EMP components: Relative number of people who belong to or aspire to elite status (e, black series) and relative per capita income of elites (epsilon, red dotted series). Smooth with loess method (span = 0.3). All data were normalized to a scale between 1–2. (TIF) [file pone.0299063.s002.tif]

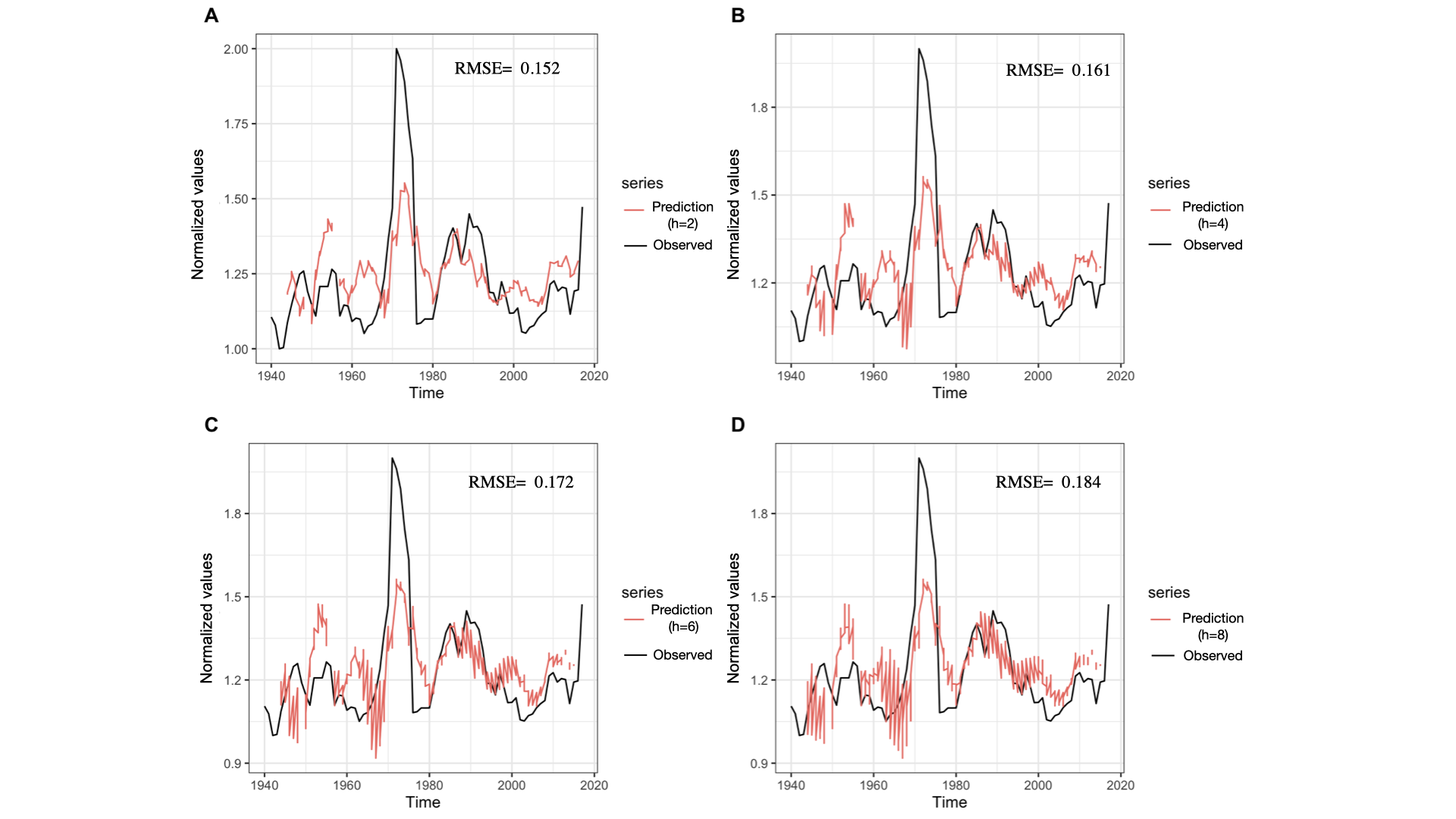

Supplement: S3 Fig — A) Two steps forward (h = 2) (RMSE = 0.152). B) Four steps forward (h = 4) (RMSE = 0.161). C) Six steps forward (h = 6) (RMSE = 0.172). D) Eight steps forward (h = 8) (RMSE = 0.184). Predicted series (RED) and Observed series (black). All data were normalized to a scale between 1–2. (TIF) [file pone.0299063.s003.tif]

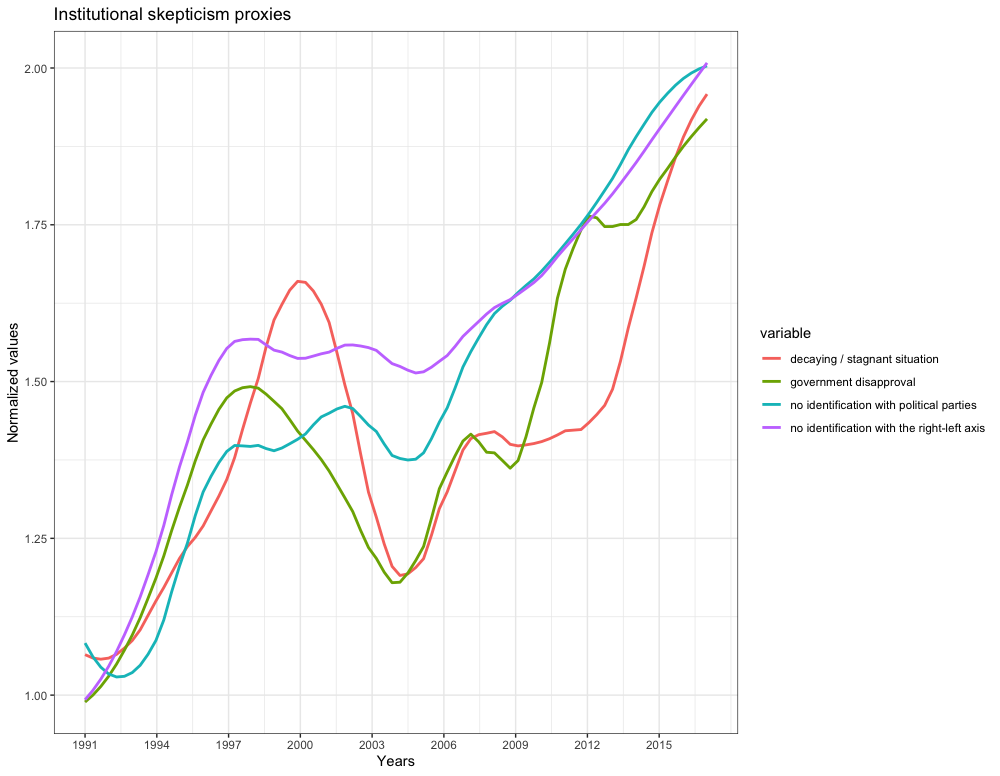

Supplement: S4 Fig — Expectations regarding the future situation of the country (stagnation/decay) (red), government disapproval (green), no identification with political parties (blue) and no identification with the left-right axis (purple). Authors’ elaboration based on data extracted from the CEP survey (1990–2018) Smooth with loess method (span = 0.3). Shaded area represents confidence interval (95%). All data were normalized to a scale between 1–2. (TIF) [file pone.0299063.s004.tif]

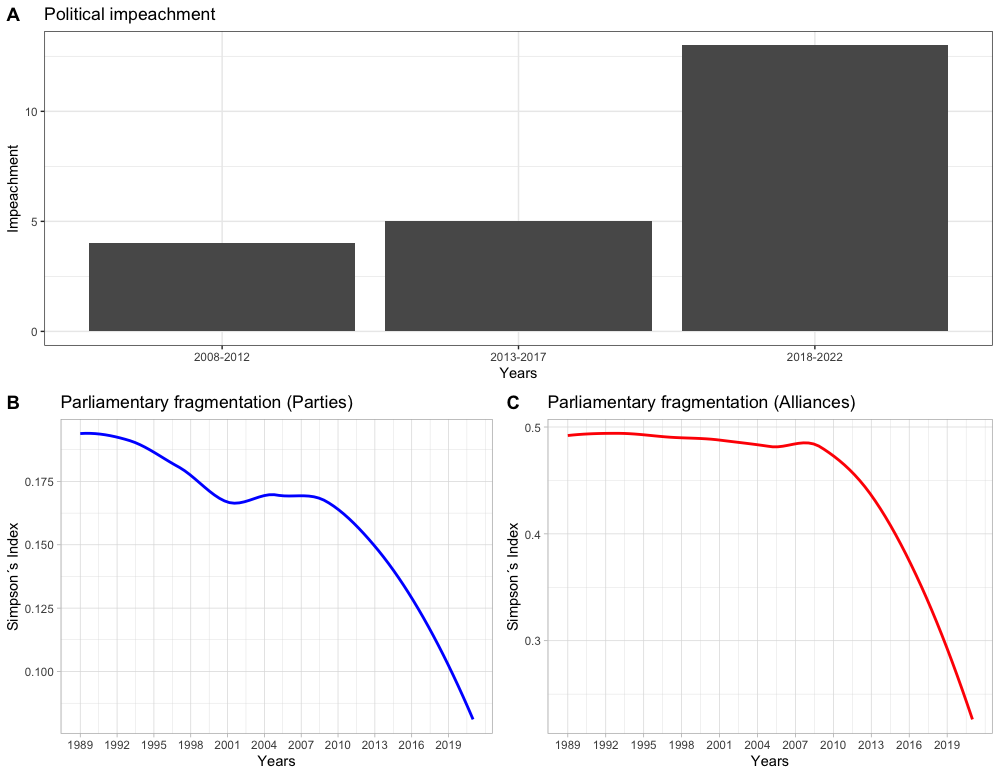

Supplement: S5 Fig — A) Dynamics of the Simpson Index (1989–2021). Probability of selecting two deputies in the lower house and that both are from the same political party. B) Dynamics of the Simpson Index (1989–2021). Probability of selecting two deputies in the lower house and that both are from the same political alliance. C) Number of impeachments initiated in the parliament. (TIF) [file pone.0299063.s005.tif]

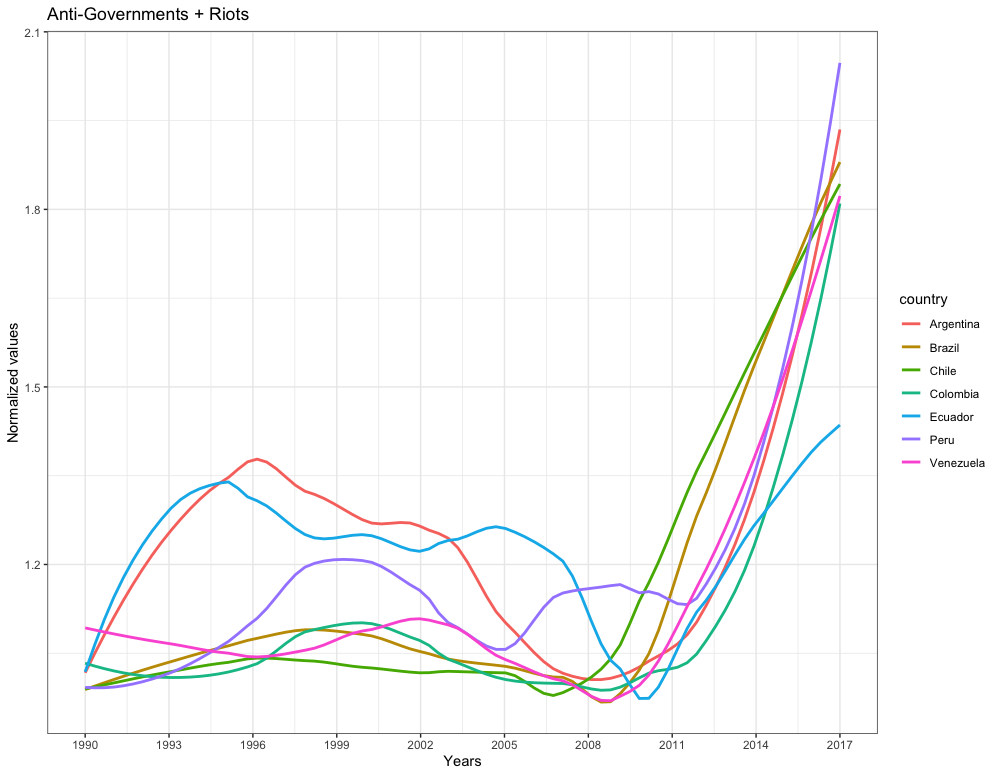

Supplement: S6 Fig — Weighted annual indicator: anti-government demonstrations (*10) + riots (*25). Data taken from Banks et al. (2021). Smooth with loess method (span = 0.3). Shaded area represents confidence interval (95%). (TIF) [file pone.0299063.s006.tif]
