# Supplementary material for: Squeezed from the top: “Social Outburst” (2019) and elite overproduction. A study of the dynamics of Chilean political instability from the approach of Structural Demographic Theory
Source: PLoS One. 2024 Jun 13;19(6):e0299063. doi: 10.1371/journal.pone.0299063 (PMC11175532; doi:10.1371/journal.pone.0299063)
Supplement: S1 Table — Analysis of the relationship with the observed political instability index. (DOCX) [file pone.0299063.s007.docx]

**S1.Table. Alternative models. Analysis of the relationship with the observed political instability index.**

| **Models** | **Parameter value** | **Pseudo-R2** | **AIC** |
| --- | --- | --- | --- |
| **SFDpb^a^xMMP^b^x. EMP^c^** | **a=0.54* ; b=0.39*; c=0.07** | **0.55** | **-67.9** |
| **SFDpb^a^ x MMP^b^** | **a=0.50*; b=0.32*** | **0.53** | **-69.2** |
| **SFDpb^a^ x EMP^b^** | **a=0.344*; b=0.32*** | **0.20** | **-39.7** |
| **MMP^a^ x EMP^b^** | **a=0.14*; b=0.43*** | **0.10** | **-26.9** |
| **MMP^a^** | **a=0.443*** | **0.02** | **-3.01** |
| **EMP^a^** | **a=0.54*** | **0.10** | **-24.97** |
| **SFDpd^a^** | **a=0.61*** | **0.14** | **-22.57** |

* Significance level (p-value<0.05)
